# Efficacy and safety of torsemide versus furosemide in heart failure patients: A systematic review of randomized controlled trials

**DOI:** 10.1002/clc.24088

**Published:** 2023-08-22

**Authors:** Huzaifa Ahmad Cheema, Saleha Azeem, Abdullah Ejaz, Faiza Khan, Anza Muhammad, Abia Shahid, Abdulqadir J. Nashwan, Muhammad Haisum Maqsood, Sourbha S. Dani, Robert J. Mentz, Marat Fudim, Gregg C. Fonarow

**Affiliations:** ^1^ Department of Cardiology King Edward Medical University Lahore Pakistan; ^2^ Department of Medicine King Edward Medical University Lahore Pakistan; ^3^ Hamad Medical Corporation Doha Qatar; ^4^ Department of Medicine Lincoln Medical Center Bronx New York USA; ^5^ Division of Cardiovascular Medicine, Beth Israel Lahey Health Lahey Hospital and Medical Center Burlington Massachusetts USA; ^6^ Division of Cardiology, Department of Medicine Duke University Medical Center Durham North Carolina USA; ^7^ Department of Medicine Duke University Medical Center Durham NC USA; ^8^ Duke Clinical Research Institute Durham North Carolina USA; ^9^ Ahmanson‐UCLA Cardiomyopathy Center, Division of Cardiology University of California Los Angeles Los Angeles California USA

To the Editor,

Heart failure (HF) is a leading cause of morbidity and mortality globally.^1^ Loop diuretics, such as torsemide and furosemide, are routinely used for managing fluid overload in patients with HF.^2^ Although furosemide is the most widely used loop diuretic, some studies and prior meta‐analyses suggested that torsemide may be superior to furosemide with fewer adverse effects while achieving similar or better outcomes including potential survival benefit.^3^, ^4^ However, current evidence remains inconsistent and previous reviews may have been biased due to the inclusion of observational studies.^3^ In addition, the results of the TRANSFORM‐HF trial by Mentz et al., the largest randomized controlled trial (RCT) on this topic to date involving 2859 patients, have recently been published.^5^ Hence, we conducted an updated systematic review of RCTs to provide more conclusive evidence regarding the efficacy and safety of torsemide versus furosemide in HF patients.

This systematic review was conducted following the *Cochrane Handbook for Systematic Reviews of Interventions* and was registered with PROSPERO (CRD42023396961). A literature search using MEDLINE (PubMed), Embase, the Cochrane Library and ClinicalTrials.gov databases was conducted from inception to February 2023 utilizing search terms related to “furosemide,” “torsemide,” “diuretics,” and “heart failure.” The inclusion criteria consisted of all RCTs comparing torsemide with furosemide in patients with HF. The data were screened and extracted by two study investigators independently. Our primary outcomes were the risk of all‐cause mortality and all‐cause hospitalization while our secondary outcomes comprised the risk of cardiac mortality and any adverse events. The risk of bias in the included studies was assessed by the revised Cochrane Risk of Bias tool (RoB 2.0). Due to the heterogeneous nature of included trials in terms of study design, sample size, patient population, and intervention characteristics, we did not conduct a meta‐analysis. We synthesized the results narratively and presented individual estimates of studies using forest plots via RevMan 5. A subgroup analysis was performed for our primary outcomes based on the setting where the diuretic therapy was initiated (in‐hospital vs. outpatient).

After applying the eligibility criteria, we included 9 RCTs (3928 patients) out of a total of 790 studies in this review.^5^, ^6^, ^7^, ^8^, ^9^, ^10^, ^11^, ^12^, ^13^ The detailed screening process is depicted in a PRISMA flowchart (Figure 1). Diuretic therapy was started in an in‐hospital setting in five studies and an outpatient setting in the remaining four. The detailed characteristics of the included RCTs are provided in Table 1. Three studies were deemed to have a high risk of bias, three had some concerns of bias, and the remaining three were at low risk of bias (Figure S1).

**Figure 1:**
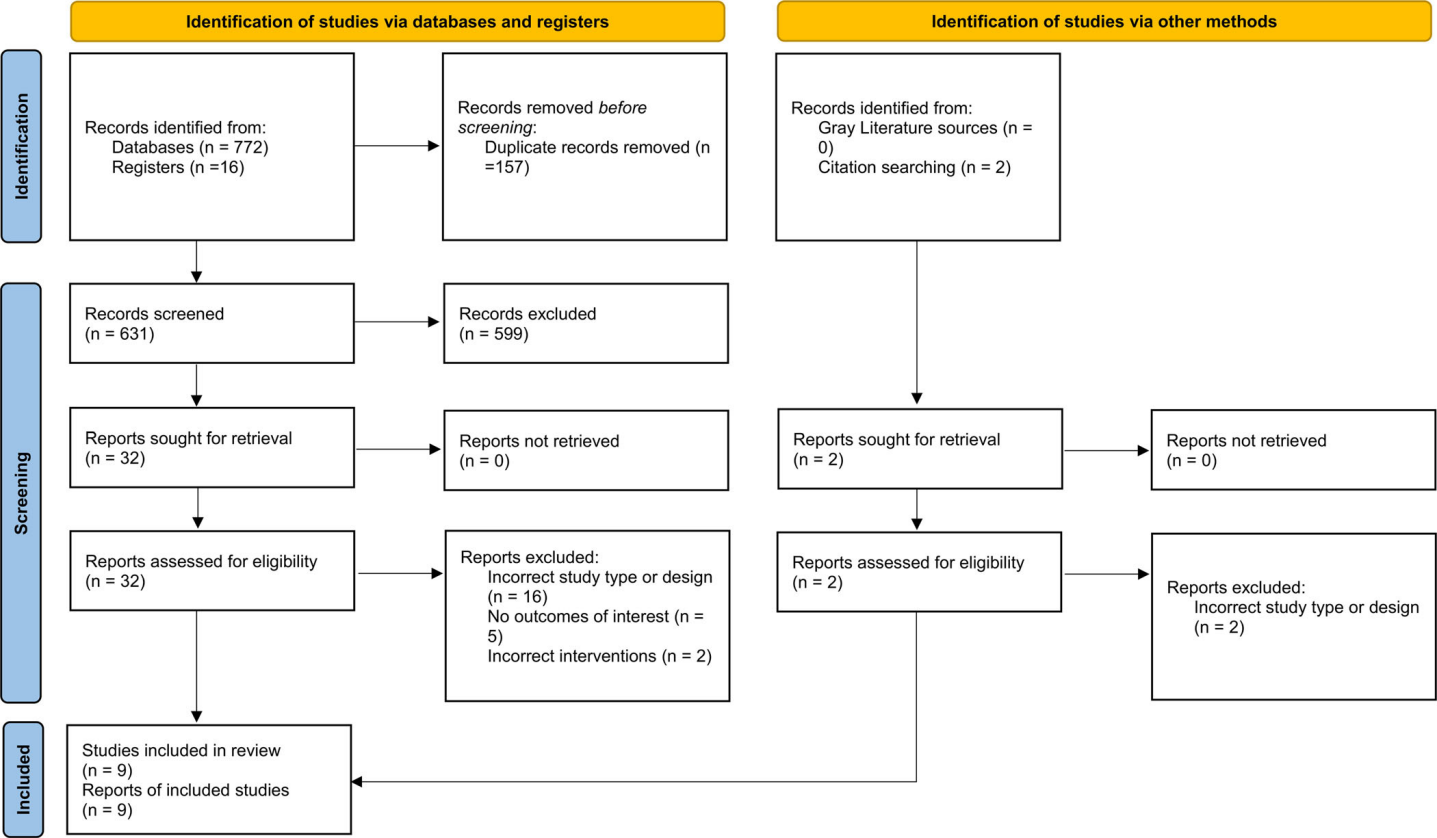
PRISMA 2020 flowchart.

**Table 1:** Characteristics of included trials.

Study ID	Country	Study setting	Study arms	No. of patients	Age (years)^a^	Male (%)	NYHA class	Intervention dosages	Follow‐up duration	Type of HF
Noe 1999	USA	Outpatients	Torsemide	103	75.1	57.3	Mean 2.34	Oral mean daily dose of 4.4 mg	6 months	Any type
			Furosemide	137	75.1	54.0	Mean 2.36	Oral mean daily dose of 10 mg		
Murray 2001	USA	Hospitalized patients	Torsemide	113	64.1 ± 10.9	49	Mean 2.8	Oral mean daily dose of 72 mg	Mean 324 days	HF with systolic dysfunction
			Furosemide	121	64.1 ± 12.4	46	Mean 2.6	Oral mean daily dose of 136 mg	Mean 318 days	
Muller 2003	Switzerland	Outpatients	Torsemide	122	74.4 ± 11.0	45.1	Mean 2.47	Daily 10 mg	9 months	Any type
			Furosemide	115	73.2 ± 10.2	40.9	Mean 2.37	Daily 40 mg		
Paterna 2005	Italy	Hospitalized patients	Torsemide	42	73.5 ± 16	64.2	Class IV	IV 200 mg twice daily	6–18 months	HF with LVEF < 35%
			Furosemide	42	74.3 ± 17	66.6		IV 500 mg twice daily		
Kasama 2006	Japan	Hospitalized patients	Torsemide	22	68 ± 6	75.0	Classes II–III	Oral 4–8 mg/day	6 months	Nonischemic HF with LVEF < 45%
			Furosemide	22	68 ± 9	70.0		Oral 20‐40 mg/day		
TORAFIC 2011	Spain	Outpatients	Torsemide	77	68.1 ± 11.4	55	Classes II–III	Oral 10‐40 mg daily	32 weeks	Any HF
			Furosemide	78	69.3 ± 9.8	62		Oral 40‐160 mg daily		
Trippel 2018	Germany	Outpatients	Torsemide	17	68 ± 8.3	76	NR	Oral 5 mg torsemide	9 months	HFpEF with T2DM
			Furosemide	18	69.3 ± 8.1	39		Oral 20 mg daily		
Balsam 2020	Poland	Hospitalized patients	Torsemide	16	74 (49–85)	68.8	Classes II–III	Mean 70 mg	3 months	Any HF
			Furosemide	24	65 (58–80)	83.3		Mean 100 mg		
Mentz 2023	USA	Hospitalized patients	Torsemide	1431	64 ± 14	65.2	NR	Mean 77.8 mg	30 months for death and 12 months for hospitalizations	Any HF
			Furosemide	1428	65 ± 14	61.0		Mean 68.4 mg		

Abbreviations: HF, heart failure; HFpEF, heart failure with preserved ejection fraction; IV, intravenous; LVEF, left ventricular ejection fraction; NR, not reported; NYHA, New York Heart Association; T2DM, Type 2 diabetes mellitus.

^a^ Data reported as mean ± SD or median (IQR).

All studies that reported data for mortality (*n* = 7) demonstrated a nonsignificant difference in the risk of all‐cause mortality between the torsemide and furosemide groups (Figure 2A). Among studies that reported data for hospitalization, only Murray et al. reported a decrease in all‐cause hospitalizations (risk ratio 0.52, 95% confidence interval: 0.32–0.85; Figure 2B) while the results of the other studies (*n* = 6), including TRANSFORM‐HF, were nonsignificant. The results were similar across studies regardless of the setting where the diuretic therapy was initiated.

**Figure 2:**
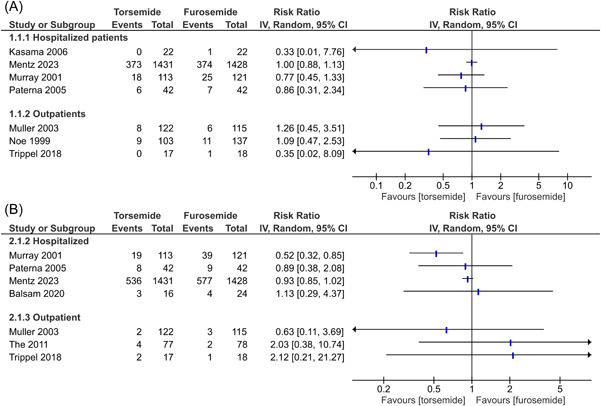
Effects of torsemide versus furosemide on: (A) all‐cause mortality; and (B) all‐cause hospitalizations in heart failure patients.

Results from three trials each showed a nonsignificant reduction in the risk of cardiac death (Figure S2). However, these studies lacked sufficient power, and the TRANSFORM‐HF trial did not report this outcome. The incidence of adverse events was comparable between the two groups in all studies except Noe et al. which showed a significant increase with torsemide (Figure S3).

In this systematic review of nine RCTs (3928 patients), the incidence of all‐cause mortality, all‐cause hospitalization, cardiac mortality and adverse events was not significantly different between patients receiving torsemide or furosemide therapy in the majority of the trials. Furthermore, these results were similar across both in‐patient and outpatient settings. However, it should be noted that most studies, except TRANSFORM‐HF, were severely underpowered for the efficacy outcomes.

The most recent RCT by Mentz et al., TRANSFORM‐HF, yielded no significant difference between the groups in all‐cause mortality or all‐cause hospitalization.^5^ In contrast, some prior meta‐analyses have shown that torsemide decreased the risk of hospitalization and/or readmissions due to HF.^3^, ^4^, ^14^ The meta‐analysis by Abraham et al. also reported a lower risk of cardiac mortality with torsemide^3^; however, this finding was primarily based on observational data, and hence, is not replicated in our synthesis of RCTs. Nevertheless, it is worth noting that there is a trend toward decreased cardiac mortality with torsemide in the trials analyzed in our review. The TRANSFORM‐HF did not report cardiac mortality; therefore, it is possible that a benefit might exist but is not seen due to a lack of power resulting from the small sample sizes of individual RCTs.

Some properties of torsemide may make it a desirable alternative to furosemide even in the context of comparable clinical outcomes. It has a longer half‐life and causes less frequent micturition than furosemide due to its increased protein‐binding capacity.^6^ Furthermore, torsemide has been reported to have beneficial effects on cardiac remodeling by inhibiting aldosterone receptors and decreasing collagen cross‐linking by decreased myocardial expression of active lysyl oxidase.^15^ Therefore, some benefits of torsemide might only become apparent in longer‐term follow‐up. The TRANSFORM‐HF biomarker sub‐study will shed some light shortly on the underlying mechanistic differences between these two diuretics.

Some limitations of our systematic review need to be considered. Due to a high degree of clinical and methodological heterogeneity in the included studies such as the trial designs, the doses of drugs used, and the severity of HF, we were unable to conduct a meta‐analysis. Furthermore, the results of the TRANSFORM‐HF trial should be contextualized within the significant rates of patient crossover and nonadherence in the two intervention groups. This might have biased its findings toward the null.

In conclusion, torsemide and furosemide are comparable regarding all‐cause and cardiac mortality, hospitalization, and adverse events in patients with HF. The choice of diuretic therapy will depend on the clinician, patient, and payer preferences but the emphasis should be on adequate dosing instead of the specific diuretic selected. Further large‐scale RCTs, with strict protocol adherence, investigating cardiac mortality, and with long‐term follow‐up are required to elucidate any potential beneficial effects of torsemide.

## CONFLICT OF INTEREST STATEMENT

Robert J. Mentz received research support and honoraria from Abbott, American Regent, Amgen, AstraZeneca, Bayer, Boehringer Ingelheim, Boston Scientific, Cytokinetics, Fast BioMedical, Gilead, Innolife, Eli Lilly, Medtronic, Medable, Merck, Novartis, Novo Nordisk, Pharmacosmos, Relypsa, Respicardia, Roche, Sanofi, Vifor, Windtree Therapeutics, and Zoll. Gregg C. Fonarow reports consulting for Abbott, Amgen, AstraZeneca, Bayer, Cytokinetics, Edwards, Eli Lilly, Johnson & Johnson, Medtronic, Merck, Novartis, and Pfizer. Other authors declare no conflict of interest.

## Supporting information

Supporting information.
