# Supplementary material for: Efficacy and safety of torsemide versus furosemide in heart failure patients: A systematic review of randomized controlled trials
Source: Clin Cardiol. 2023 Aug 22;47(1):e24088. doi: 10.1002/clc.24088 (PMC10765996; doi:10.1002/clc.24088)
Supplement: Supplementary file 1 — Supporting information. [file CLC-47-e24088-s001.docx]

**Supplementary Figure 1.** Quality assessment of included trials.


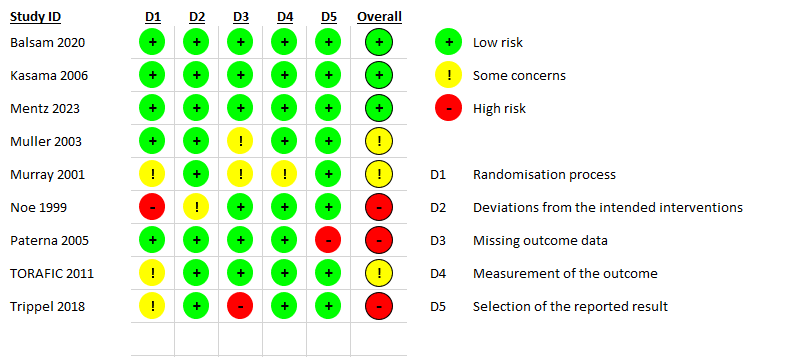


**Supplementary Figure 2. Effect of torsemide vs furosemide on cardiac mortality in patients with heart failure.**

**
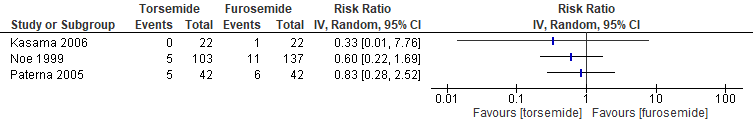
**

**Supplementary Figure 3. Effect of torsemide vs furosemide on adverse events in patients with heart failure.**

**
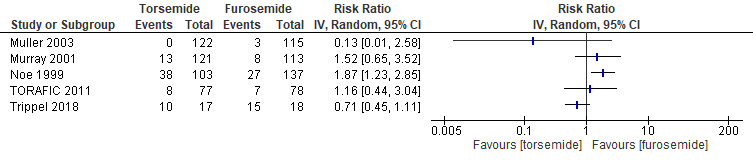
**
